# AI-Assisted ISP and Chip-Off Forensic Framework for Damaged Android Devices

**DOI:** 10.3390/s26123639

**Published:** 2026-06-07

**Authors:** Leila Rzayeva, Aigerim Alibek, Altynbay Abdykassym, Murat Zhakenov

**Affiliations:** 1 Research and Innovation Center “CyberTech”, Astana IT University, Astana 010000, Kazakhstan; l.rzayeva@astanait.edu.kz (L.R.); muratzhakenov@outlook.com (M.Z.); 2 Digital Heritage of Eurasia LLP, Astana 010000, Kazakhstan; altinbai1955@gmail.com

**Keywords:** mobile forensics, android forensics, Chip-Off extraction, In-System Programming (ISP), damaged mobile devices, embedded memory analysis, UFS memory, eMMC memory, digital forensic extraction

## Abstract

Physical damage to smartphones creates a persistent bottleneck in mobile forensic practice: once a device can no longer be accessed through its operating system, conventional logical acquisition fails, and investigators face a choice between accepting data loss and escalating to hardware-level intervention. This paper describes an integrated forensic workflow that addresses this gap by combining In-System Programming (ISP) and Chip-Off memory extraction with an AI-assisted artifact localization and prioritization layer. The workflow was evaluated on 18 physically damaged Android smartphones for which all standard acquisition paths were unavailable. Hardware extraction produced verified binary memory images from all 18 devices. A 1D-CNN localization classifier subsequently screened those images, achieving F1-score = 0.88 and ROC-AUC = 0.94 on the synthetic test partition. Prioritization of candidate windows reduced manual review volume by 78%, cut total expert review time by 63%, and shortened the time to first relevant artifact from 42 to 14 min relative to unassisted examination (indicative estimates based on three examiner sessions; no inferential statistical test was performed). The study contributes a formalized, criteria-driven decision model for selecting between ISP and Chip-Off, which are experimentally validated thermal extraction profiles for eMMC, UFS, and PoP/RAM memory.

## 1. Introduction

Smartphones are now among the most consequential sources of evidence in criminal investigations. They continuously log calls, messages, location history, financial transactions, and social interactions, making them valuable to investigators and, by extension, to the integrity of legal proceedings [1,2]. The global proliferation of smartphone use has driven mobile forensics to become one of the most technically demanding and fastest-evolving branches of digital investigation [1,2]. Extracting evidence from smartphones is considerably more complex than from conventional computers. Tight hardware–software integration, embedded eMMC or UFS storage, full-disk and file-based encryption, secure boot chains, and manufacturer-enforced access controls all constrain what standard logical tools can obtain [1,2,3]. These difficulties intensify when a device has sustained physical damage: a destroyed screen, liquid ingress, burned motherboard circuitry, or even a forgotten PIN can render logical acquisition entirely infeasible [2,3,4]. Hardware-level extraction—principally ISP and Chip-Off—was developed specifically to address this gap. ISP establishes a low-level programmer connection directly to the memory interface without chip removal; Chip-Off involves desoldering and externally reading the memory package. Both methods have demonstrated practical utility but require specialized equipment, controlled thermal procedures, and substantial post-processing effort [4,5,6]. Crucially, both produce raw binary images rather than parsed file systems, making the evidentiary yield of a successful acquisition dependent on what can subsequently be extracted from the dump. Artificial intelligence offers a path toward more efficient post-extraction analysis. Recent surveys document growing interest in AI-assisted forensic workflows [7,8,9], yet the literature on combining hardware image acquisition with machine-learning-based artifact localization remains sparse, particularly for damaged-device scenarios. Practical deployment is further constrained by dataset limitations, practitioner skill requirements, and workflow integration challenges [10,11,12,13,14]. Low-level recovery from damaged Android devices can itself be limited by storage-level deletion mechanisms such as TRIM [15], underscoring the need for a workflow that addresses both acquisition and analysis in a unified, reproducible manner. This study presents an integrated forensic workflow combining ISP and Chip-Off extraction with AI-assisted artifact localization and prioritization for physically damaged Android devices. The selection between ISP and Chip-Off is formalized through measurable hardware criteria.

## 2. Materials and Methods

### 2.1. Methods of Information Extraction and Classification of Mobile Device Faults

**Figure 1:**
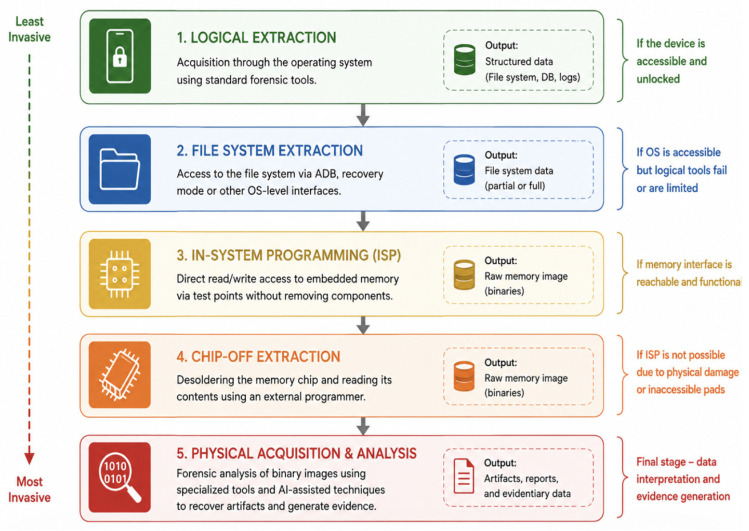
Hierarchy of extraction methods.

Working with physically damaged mobile devices requires a disciplined approach that avoids compounding the damage already present. The strategy adopted in this study proceeds through successive levels of intervention, beginning with visual inspection and electrical assessment, continuing with logical access where feasible, then with ISP if the memory interface is reachable, and escalating to Chip-Off only when less invasive options cannot produce a readable dump. This ordering reflects the established hierarchy of mobile forensic extraction methods (Figure 1) [16], under which progression to more destructive procedures is undertaken solely when less intrusive approaches have been ruled out on technical grounds.

### 2.2. Formalization of the Minimal Invasiveness Principle

Rather than leaving the choice between ISP and Chip-Off to individual judgment, the workflow formalizes it around a set of observable hardware indicators. Each device was evaluated against measurable electrical and physical criteria before any extraction attempt was initiated, with the explicit goal of preserving board integrity while still obtaining a readable memory image.

The following indicators were evaluated during the diagnostic stage:

- Stability of current consumption during power-on attempts;
- Accessibility and physical condition of ISP pads;
- Presence of short circuits in memory power lines;
- Signal integrity during low-level communication attempts;
- Degree of corrosion or liquid-induced oxidation;
- Thermal stability of the motherboard during localized heating;
- Accessibility of memory-related test points;
- Physical condition of solder pads and surrounding PCB layers.

ISP was attempted first whenever the memory package was electrically reachable and the programmer could establish stable communication without requiring chip removal. Chip-Off was reserved for situations where this was not achievable, typically because corrosion had advanced too far, signal lines were unreliable, test points were physically obstructed, pad geometry was compromised, or short-circuit conditions made non-destructive contact unsafe.

By grounding the selection in concrete, observable criteria rather than experience-based intuition alone, the model reduced the incidence of unnecessary Chip-Off attempts and lowered the probability of irreversible thermal or mechanical damage to memory components. It also improved procedural reproducibility: operators following the same diagnostic checklist arrived at consistent extraction decisions, which is important for evidentiary traceability. The decision criteria used to determine whether ISP or Chip-Off extraction should be applied are summarized in Table 1.

**Figure 2:**
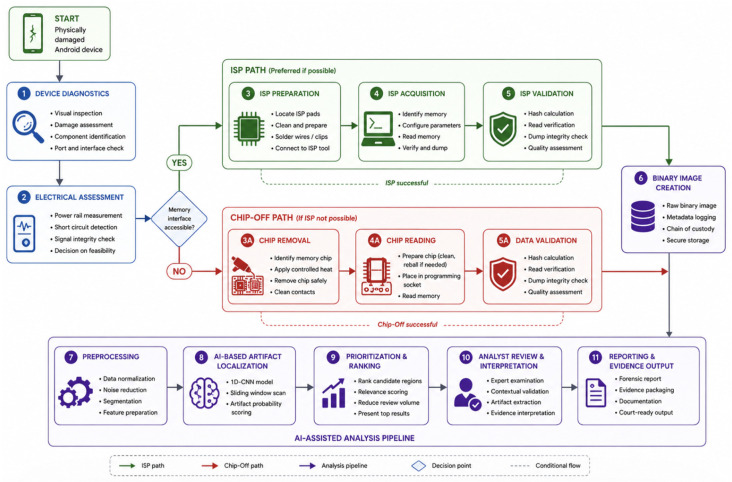
Combined method of information extraction.

According to the developed combined method (Figure 2), the workflow begins with the initial diagnosis of the device. Where possible, measurable thresholds were defined for each criterion: current stability is assessed as consumption variance ≤5 mA over a 30 s observation window; corrosion severity is rated on a three-point scale (low: <10% pad area affected, moderate: 10–30%, severe: >30%); and signal quality is evaluated by counting communication retry failures (ISP preferred: zero retries; Chip-Off indicated: ≥3 consecutive failures).

At this stage, external damage to the case and display, traces of impacts, and overheating or liquid ingress are recorded; the device’s response to the power source is assessed, as well as the condition of the connectors, battery and motherboard. If the device shows signs of functionality—such as vibration or audio response—but does not display an image, then logical extraction via USB/ADB/EDL without direct intervention at the board level is considered the initial scenario. If the device does not respond to power supply or buttons, electrical diagnostics is performed: the current consumption of the laboratory source, and the resistance in the memory power lines are measured, and the diodes of the signal lines are checked. If the configuration is stable and the test sites are protected, a slow attempt is made to read the data through the ISP. In case of a short circuit, unstable connection, severe corrosion, connection failures or inability to access the contacts of the chip, Chip-Off extraction is selected as the primary access method. For liquid-damaged devices, the board was cleaned and dried before repeated electrical assessment, after which another electrical check was carried out. For locked devices, the availability of backup- or cloud-based recovery options was evaluated first, and hardware intervention at the chip level was mainly used to save the memory dump and confirm its authenticity. The classification of errors in the study was based on the technical characteristics that determine the choice of the data access method. The main categories were as follows: power-supply and boot-circuit failures, which manifest themselves in a complete lack of reaction to power on; cyclic reset or unstable power supply; interface and optical defects such as black screen, sensor failure and damage to the display channel; liquid-exposure damage characterized by oxidation traces, corrosion on contact surfaces and possible short circuits; mechanical damage to the motherboard and chip housings; as well as software failures such as password, PIN, or update errors. This classification enabled systematic selection of the appropriate access method and reduced the risk of unnecessary chip removal in cases where logical access or ISP extraction was sufficient.

From the point of view of hardware implementation, the study primarily considered devices with embedded eMMC and UFS memory, since these standards are the most prevalent in the Android ecosystem and require distinct conditions for desoldering, cleaning, and readout. To identify the memory type, each device was opened and the chip marking read directly under a microscope-device model information alone was considered insufficient, particularly for units that may have undergone prior repair or component replacement. eMMC packages are characterized by square BGA housings, while UFS packages are typically rectangular and high-speed. A memory-type-specific thermal profile was applied at each extraction; the full experimental basis for these profiles is described in Section 2.3.

### 2.3. Experimental Validation of Thermal Profiles

The temperature ranges applied during chip removal were not adopted arbitrarily. A series of controlled extraction trials was conducted across all three memory types to establish which thermal parameters produced reliable results without damaging the contact pads or the PCB substrate. The objective was to map the relationship between thermal exposure and extraction outcome across three dimensions: successful chip removal rate, pad-damage incidence, and post-extraction read stability.

Each trial varied one or more parameters—preheating temperature, peak localized heat, and airflow rate—while holding the others constant. After every attempt, the board and chip were examined under a microscope to document solder joint quality, pad geometry, and any signs of substrate stress or deformation.

Trials using temperatures below the recommended range consistently produced incomplete solder reflow, leaving the chip partially bonded and prone to mechanical tearing during removal—a failure mode that in several cases caused contact pad detachment. At the other extreme, overheating caused PCB substrate warping, layer delamination, and pad lift-off—damage that was irreversible and in affected devices rendered the chip unreadable.

The profiles reported here represent the parameter combinations that yielded the best ratio of successful extractions to pad damage across all trials. For eMMC chips, preheating to 150–170 °C with localized heat of 380–410 °C produced stable results with low incidence of pad damage. UFS packages, which are denser and more thermally demanding, required preheating to 170–200 °C and localized peaks of 420–450 °C. PoP/RAM configurations required the most conservative treatment-staged heating with reduced airflow, given their elevated risk of interlayer separation under sustained thermal pressure. The optimized thermal extraction profiles identified for each memory type are summarized in Table 2.

As shown in Table 3, these results make it clear that treating eMMC, UFS, and PoP/RAM as thermally interchangeable is not defensible forensic practice. The differences in optimal profiles are large enough that applying one memory type’s parameters to another meaningfully raises the risk of extraction failure and, by extension, irretrievable evidence loss.

Before any chip was removed, the board was fully exposed by taking off the SIM tray, rear cover, battery, and shielding cans. Adjacent components were covered with thermal patch material to limit heat spread; flux was applied along the chip package edges to promote solder reflow; and the board was brought to preheating temperature gradually before the localized heat source was applied. After desoldering, the chip was lifted carefully, cooled, cleaned under a microscope, and transferred to the programmer for readout.

Regardless of the chosen method, after confirming the access channel, critical memory areas were read, including boot partitions, EXT_CSD, or LUN areas, and then a complete user memory image was generated. To verify the integrity of the dump, key areas were reread and the results were compared. This approach made it possible to increase the reproducibility of the procedure and reduce the likelihood of using a partially damaged or unstable image in further forensic analysis. In general, the proposed methodology combines engineering diagnostics, controlled hardware extraction and step-by-step verification, which makes it applicable for the study of both partially functional and completely inactive mobile devices.

The experimental sample included Android smartphones manufactured by Samsung, Huawei, and OPPO, which differ in the design features of the motherboard, the type of internal memory, and the level of hardware protection. The study included both devices with limited access and physically damaged and completely inoperable devices. The general characteristics of the studied devices and their selection criteria are given in Table 4.

Table 4 lists each of the 18 devices individually. The sample comprised 14 eMMC devices and four UFS devices, totalling 18 units across three manufacturers. The sample was intentionally heterogeneous: it encompasses three manufacturers, two primary memory standards (eMMC and UFS), and five damage categories (water exposure, impact damage, short circuit, PIN/password lock, and boot failure). This diversity was deliberate—it allowed the workflow to be evaluated across the range of conditions that forensic practitioners actually encounter, rather than being optimized for a single hardware profile. Device models, memory types, applied extraction methods, and acquisition outcomes are reported per device, providing sufficient detail for independent reproducibility assessment.

### 2.4. Materials

The study used Android-based mobile devices with eMMC and UFS memory chips, as well as laboratory devices designed for hardware extraction; the study also prepared the chips for reading. Among the tools applied are an air thermal soldering station, a microscope with a magnification of at least 10×, an antistatic mat and a bracelet, flux, 99.9% isopropyl alcohol, a thermal patch and thermal protection products for the board, tweezers, soldering, copper braid, BGA templates and a chip reading programmer for ISP operations; EASY JTAG was used with a four-bit ISP Rev 2.1 adapter, and the interaction with the chip was performed using UFPI software (v2.14). This equipment made it possible both to securely erase the memory and to read the chip directly, without removing it in cases where the hardware configuration of the device allowed it.

EMMC and UFS memory chips, which are the most typical for Android devices, were considered the main objects of hardware-oriented mining. In addition, the document considers NAND and PoP/RAM as related types of memory, which are important for identifying the chip and selecting the correct thermal profile. The main types of memory considered in the study are presented in Table 5.

As Table 5 shows, eMMC and UFS constituted the primary objects of study because they account for the overwhelming majority of internal storage in current Android devices and require fundamentally different extraction conditions. NAND and PoP/RAM were included for completeness—primarily to inform correct thermal profile selection and to cover edge cases encountered in the sample—but neither drove the core extraction protocol.

**Figure 3:**
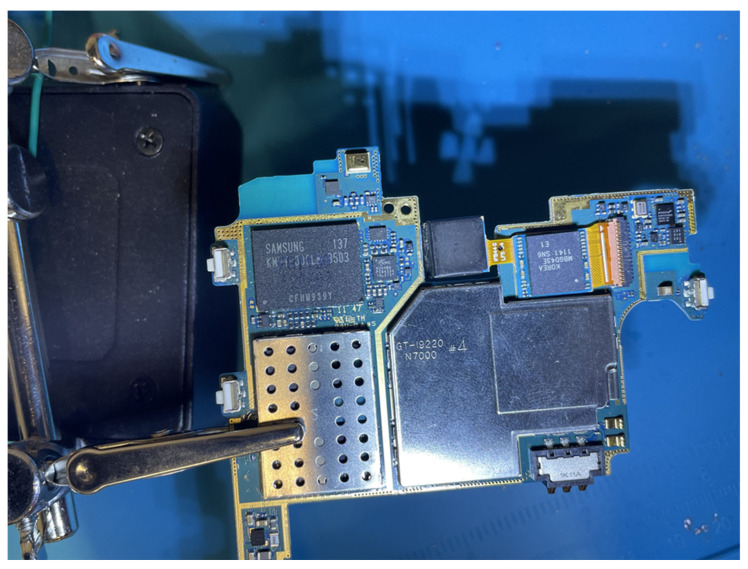
Memory chip marking.

Device model information alone was insufficient to confirm memory type, particularly for damaged units where the board may have been repaired or components replaced. Reliable identification required opening each device and reading the chip marking directly under a microscope before any extraction decision was made. For example, an eMMC chip bearing the marking “KMN…” was identified on one device motherboard, confirming its memory type and enabling correct thermal profile selection for subsequent hardware acquisition (Figure 3).

## 3. Results

### 3.1. Practical Validation of Hardware Data Extraction

**Figure 4:**
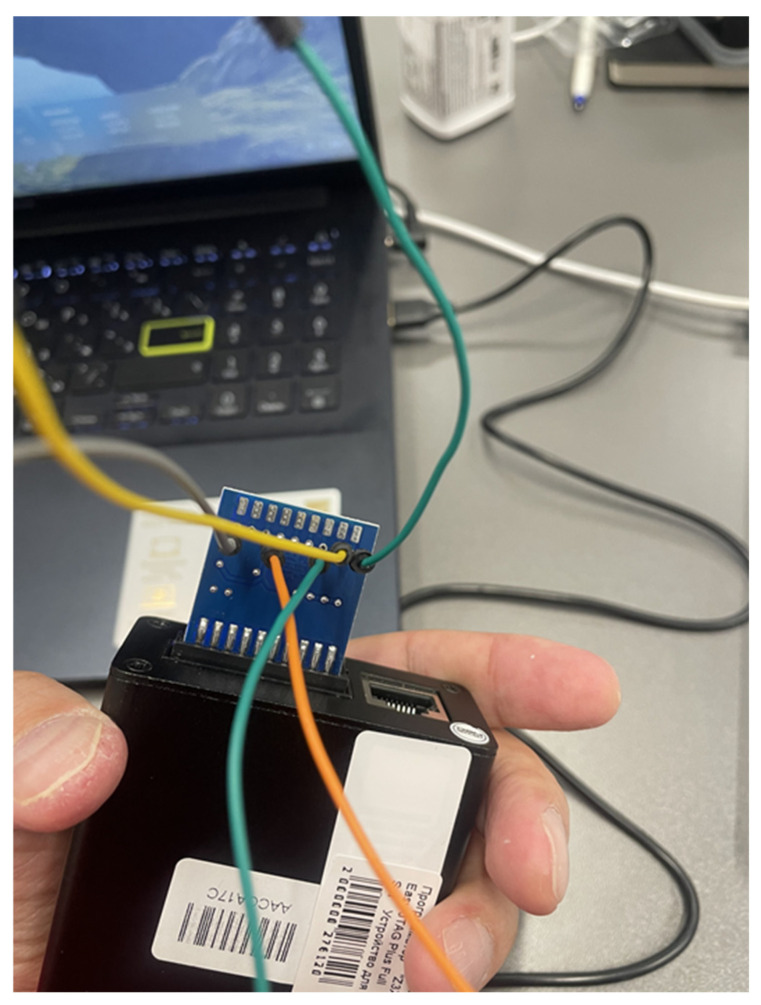
Process of connecting to the chip pins.

All 18 devices entered the study because conventional acquisition had already failed or was clearly infeasible. The reasons varied: some would not power on at all, others powered on but could not boot, and several exhibited motherboard damage severe enough to rule out any software-based approach from the outset. In each case, the workflow started at the hardware level, assessing the physical state of the board and determining whether low-level memory access was possible before any extraction was attempted. For each device, visual inspection was performed first, after which the SIM tray was removed and the enclosure was heated in a controlled manner. Heating was applied around the perimeter using circular motions at a nozzle distance of 8–12 cm, with an air temperature of 280–350 °C and a surface temperature of 70–90 °C monitored continuously with an infrared thermometer. After the adhesive softened, the rear cover was removed and the battery disconnected, providing safe access to the motherboard. ISP pad identification followed immediately (Figure 4).

**Figure 5:**
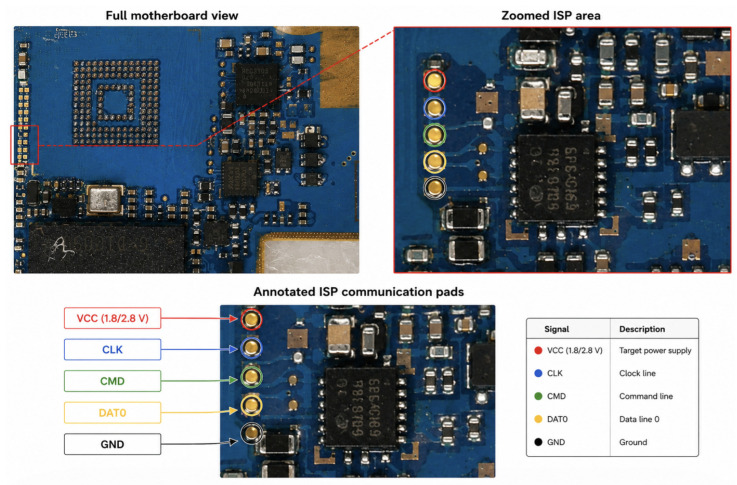
Identification of ISP communication pads on the motherboard.

After the board was exposed and cleaned, ISP communication pads were located under the microscope (Figure 5). Their physical condition was assessed against the diagnostic criteria defined in Section 2.2 to determine whether a direct programmer connection was viable without chip removal.

**Figure 6:**
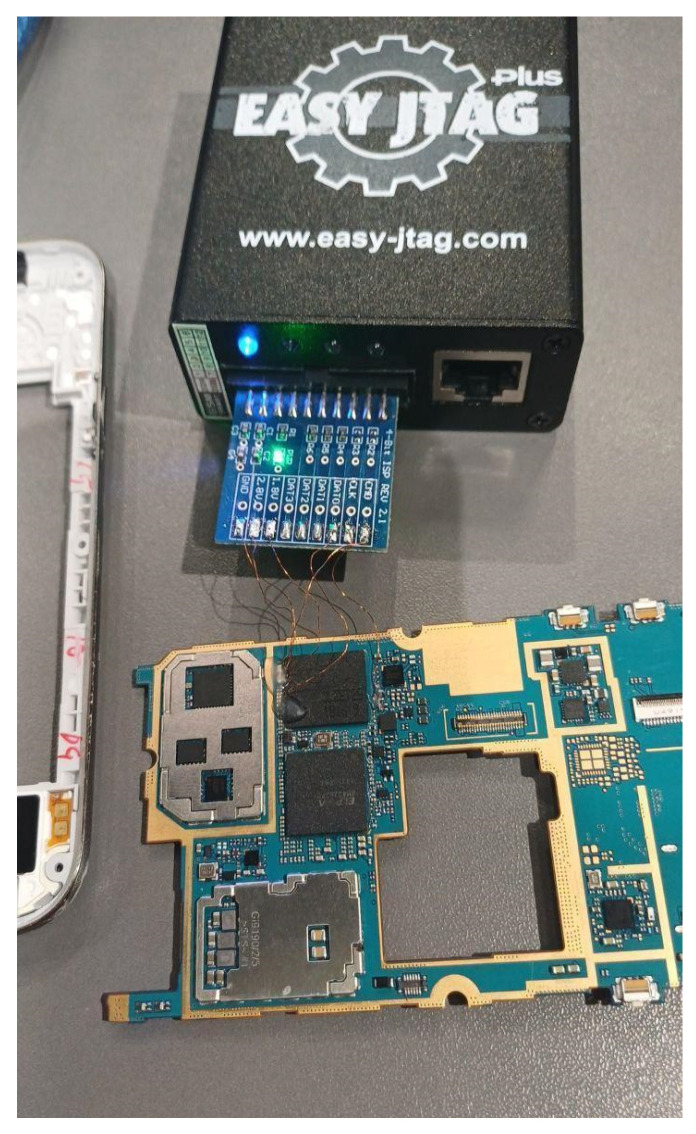
Connection of EasyJTAG programmer to ISP lines during low-level acquisition.

Where ISP pads were accessible and electrically intact, the EasyJTAG programmer was connected to the corresponding signal lines. UFPI software (version 2.14) was configured in read-only acquisition mode before any communication was initiated, ensuring that no write operations could occur during the dump session (Figure 6).

**Figure 7:**
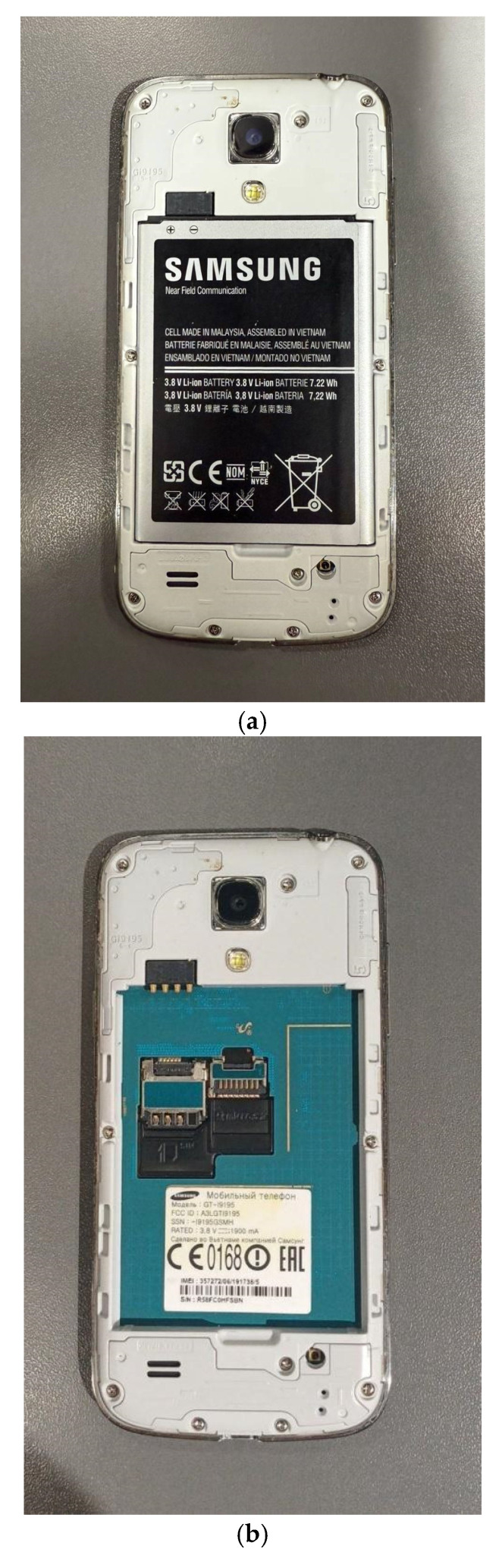

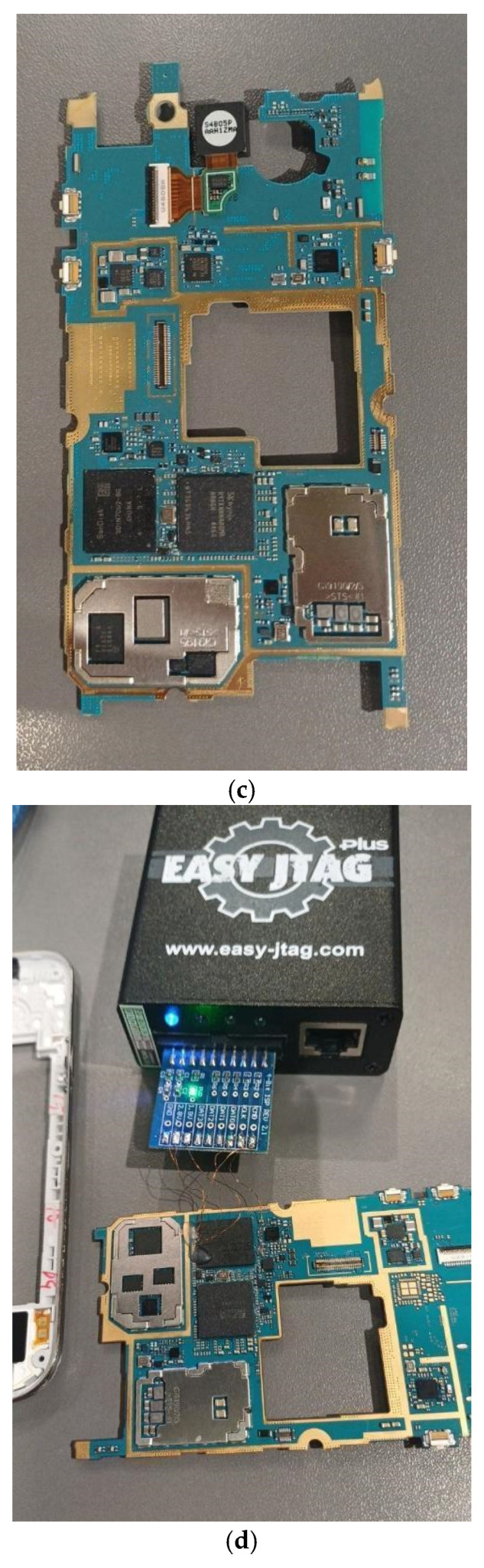
Progressive disassembly workflow of the damaged Android device before ISP-based acquisition: (**a**) rear-cover removal, (**b**) battery isolation, (**c**) motherboard extraction, (**d**) prepared board for low-level forensic access.

Connecting at the ISP level allowed memory to be read without removing the chip in cases where signal lines and power circuits remained intact, preserving the board in its original state for subsequent examination if needed. Each stage of the disassembly and connection process—cover removal, battery isolation, board extraction, pad identification, and programmer attachment—was documented with timestamps and photographs to support procedural reproducibility and chain-of-custody traceability (Figure 7).

The documented sequence makes clear that what might appear to be a preparatory phase—board inspection, disassembly, and pad identification—is in fact a complete acquisition path in its own right when ISP contact proves stable. Several of the 18 devices were fully imaged via ISP without requiring chip removal. For the remaining devices, where ISP contact was not achievable, Chip-Off was performed following the validated thermal profiles described in Section 2.3. Binary memory dumps ranging from 32 GB to 256 GB were successfully acquired across all 18 devices. In practice, however, read success depended not only on connection accuracy but also on the physical condition of the memory package and its signal lines—a finding that reinforces the importance of the pre-diagnostic stage. ISP thus functioned as a critical bridge between the hardware extraction stage and the subsequent AI-assisted analysis of the acquired memory image.

### 3.2. Forensic Integrity and Repeatability Validation

The technical success of an extraction is a necessary but not sufficient condition for forensic use—the resulting image must also be demonstrably unaltered and producible under equivalent conditions. Given that damaged devices introduce risks of partial reads and electrical interruptions, the workflow incorporated specific integrity controls at each stage rather than relying on post hoc verification alone.

Every binary image produced—whether via ISP or Chip-Off—was hashed with both SHA-256 and MD5 immediately after acquisition using dc3dd7.2, which computes hashes in a single read pass. The same critical regions (boot partition, EXT_CSD, LUN0 user data area) were then reread and re-hashed to verify that repeated access introduced no modification. Of the 18 acquisitions, 15 produced matching hashes on the first two passes; two required a third read after which all hashes matched; and one device yielded a stable but unrepeatable hash due to progressive read degradation, which was documented separately with a note that affected sectors may contain read-induced artifacts.

All physical work was performed on an ESD-protected bench with grounded mats and wrist straps throughout. Every step—thermal exposure, chip handling, ISP soldering, memory readout, and dump generation—was entered into a timestamped acquisition log with an operator identifier, so that the full sequence of physical actions could be reconstructed from the record. Logged parameters included the extraction method, thermal profile applied, heating duration, observed board condition at each stage, programmer configuration, and hash verification outcomes.

For eight of the 18 devices where hardware access remained stable after the initial acquisition, a repeat read was performed at least 48 h later under identical connection conditions. In all eight cases, the SHA-256 hash of the repeat dump matched that of the original, confirming that the ISP-based acquisition path does not alter memory content between sessions under stable hardware conditions. Chip-Off cases involving reballing were classified as non-repeatable assessments per the terminology of Cuomo et al. [2], given the irreversible physical changes involved.

The integrity-control procedures described above were designed in alignment with NIST SP 800–101 Rev. 1 [3] and ISO/IEC 27037:2012 [17], both of which require contemporaneous logging, hash-based verification, and write-blocking where technically feasible. For ISP connections via UFPI, the software was configured in read-only mode before any communication was initiated, satisfying the write-blocking requirement at the software level.

### 3.3. Results of ISP/Chip-Off Path Selection

**Figure 8:**
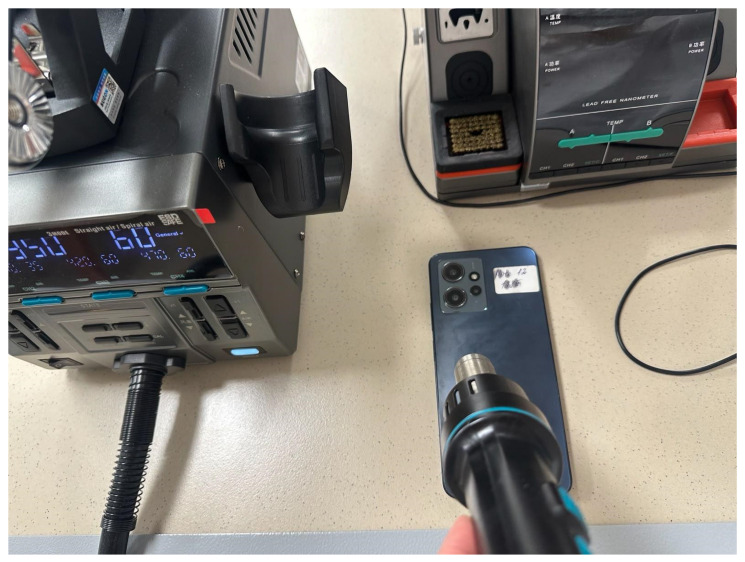
Warming up the Oppo phone case.

The results showed that it is impossible to recover data from damaged Android devices according to a single scenario applicable to all storage types. The experimental study demonstrated that each memory architecture requires a separate extraction and handling strategy due to differences in electrical organization, substrate design, thermal stability, and mechanical resistance during disassembly (Figure 8).

**Figure 9:**
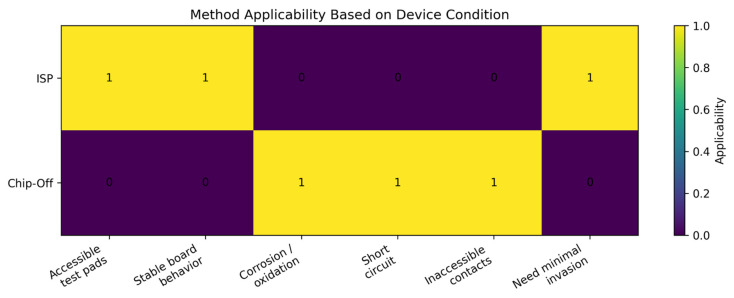
Decision logic for selecting ISP or Chip-Off based on board condition and memory accessibility.

The extraction parameters and procedures summarized here were obtained during repeated practical recovery operations on devices with eMMC, UFS, and PoP/RAM memory. UFS devices consistently required more careful handling than eMMC—higher thermal sensitivity, denser contact geometry, and greater risk of interlayer separation under sustained heat all demanded closer attention at each procedural step. PoP/RAM configurations were the most demanding of the three, due to multilayer stacking and the elevated probability of interlayer separation under thermal pressure. The experimental basis for the thermal profiles applied in each case—including the trial data, outcome metrics, and justification for the selected temperature ranges—is detailed in Section 2.3. The decision logic for selecting ISP or Chip-Off based on board condition is illustrated in Figure 9.

The comparative data in Table 6 confirm the complementary nature of the two methods. ISP achieved a 72% success rate with low physical invasiveness and an average extraction time of 1.5 h, but was limited to devices where the board condition permitted stable programmer contact. Chip-Off reached an 88% success rate at the cost of greater thermal exposure, higher pad-damage risk, and an average time of 3.8 h. These figures support the progressive escalation strategy: ISP is used as the default where the diagnostic criteria in Section 2.2 are met, and Chip-Off is reserved for cases where ISP is not viable. Neither method universally dominates; the two are best understood as complementary stages of a single adaptive workflow rather than competing alternatives.

### 3.4. Results for eMMC and UFS Handling Profiles

**Figure 10:**
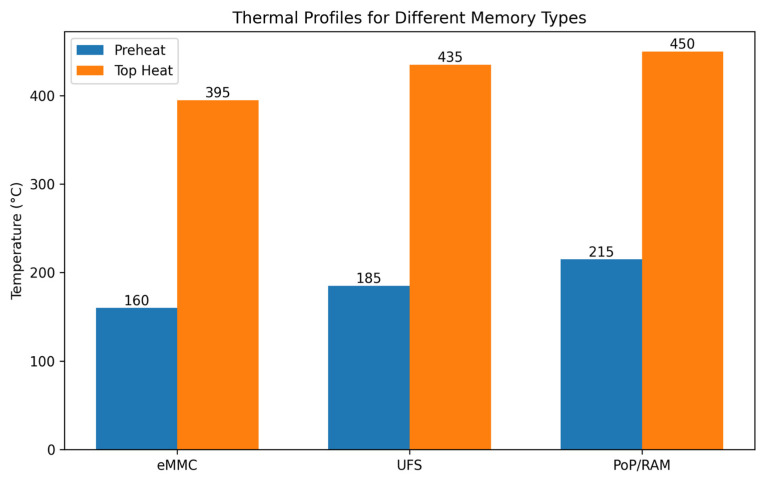
Comparison of thermal handling profiles for different memory types used in damaged Android devices.

eMMC and UFS memory types required distinct procedural approaches at every stage, not only thermally. For eMMC devices, the primary objectives were chip identification, ISP contact attempt, chip removal, if necessary, and pad preparation for readout. UFS devices demanded more care throughout: staged heating, careful flux application, controlled chip lift, and microscopic cleaning of the contact array after removal. The experimentally validated thermal profiles—eMMC: preheat 150–170 °C/peak 380–410 °C; UFS: preheat 170–200 °C/peak 420–450 °C; PoP/RAM: preheat 200–230 °C/peak 430–470 °C with reduced airflow—are fully detailed in Section 2.3 and summarized in Figure 10. The practical implication is that treating eMMC and UFS as thermally interchangeable raises the probability of chip damage to an unacceptable level. Getting the thermal and procedural parameters right at this stage is what makes the downstream analysis meaningful.

### 3.5. Relevance of Hardware Extraction Results for AI-Assisted Artifact Analysis

**Figure 11:**
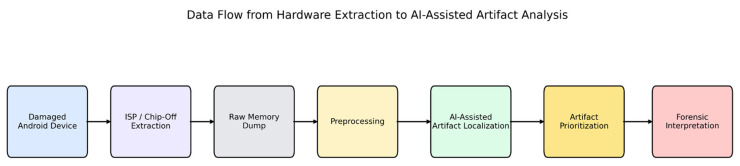
Data flow from hardware extraction to AI-assisted artifact analysis.

The binary image produced at the hardware stage is the direct input to the AI-assisted analytical layer. Once the read channel was confirmed stable—by verifying boot partition readability, EXT_CSD response, and LUN structure integrity—the full user data partition was imaged and passed to the localization module. The quality and completeness of this hardware acquisition directly determine what the analysis stage has to work with a verified, hash-confirmed image enables high-confidence artifact localization, while a degraded or partially unreadable image limits what can be recovered. The hardware-oriented stage and the AI-assisted post-processing together form a single coherent pipeline (Figure 11), and neither is substitutable for the other.

### 3.6. Main Experimental Conclusions

The totality of the achieved results allows us to draw several important conclusions. First of all, the integrated use of ISP and Chip-Off for damaged Android devices is practically justified, since the condition of the board and the presence of low-level interfaces directly determine the acceptable memory access strategy. Secondly, the success of material extraction depends on the correct identification of the type of storage device and strict compliance with specialized thermal profiles for eMMC and UFS. Thirdly, the confirmation of low-level interaction with memory and the successful preparation of the chip for external reading show that the proposed workflow leads to a practically applicable result, to obtain a solid foundation for the formation of a binary memory image. Finally, this image directly contributes to the placement and interpretation of digital artifacts based on artificial intelligence, which conceptually and technically aligns the proposed approach with the tasks of modern mobile forensics.

### 3.7. AI-Assisted Artifact Localization Performance

**Figure 12:**
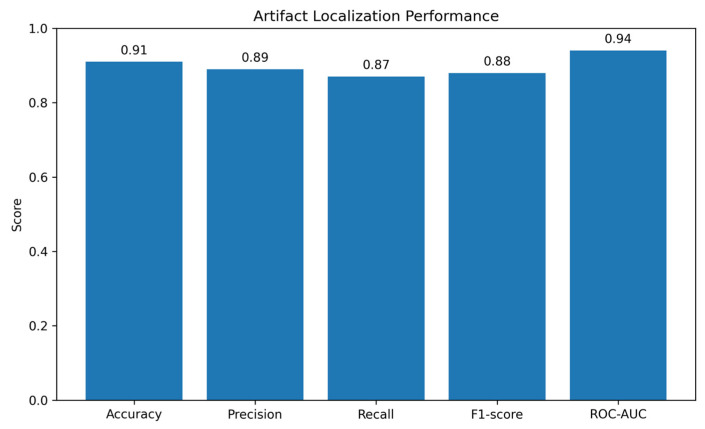
Artifact localization performance of the AI-assisted module.

After forming a binary memory image, the AI-assisted module was used to locate areas potentially containing forensic artifacts. The prototype was evaluated at the level of binary classification of memory windows into two classes: artifact-bearing and background regions. On the held-out test set (15,000 windows from three devices unseen during training), the localization module achieved accuracy = 0.91, precision = 0.89, recall = 0.87, F1-score = 0.88, and ROC-AUC = 0.94 (Figure 12). It should be noted that these metrics reflect performance on the synthetic test partition and do not directly represent expected performance on real damaged-device dumps, which may exhibit different noise profiles, encryption effects, and corruption patterns. These results should be interpreted as proof-of-concept indicators rather than operational performance guarantees.

This performance is especially important in conditions of damaged Android devices, where raw dumps may contain large amounts of irrelevant data, fragmented structures, and partially degraded storage areas. Therefore, AI-assisted localization can be regarded as a practically useful post-processing layer, reducing the amount of manual analysis and increasing the likelihood of detecting significant digital artifacts in the early stages of forensic research.

**Figure 13:**
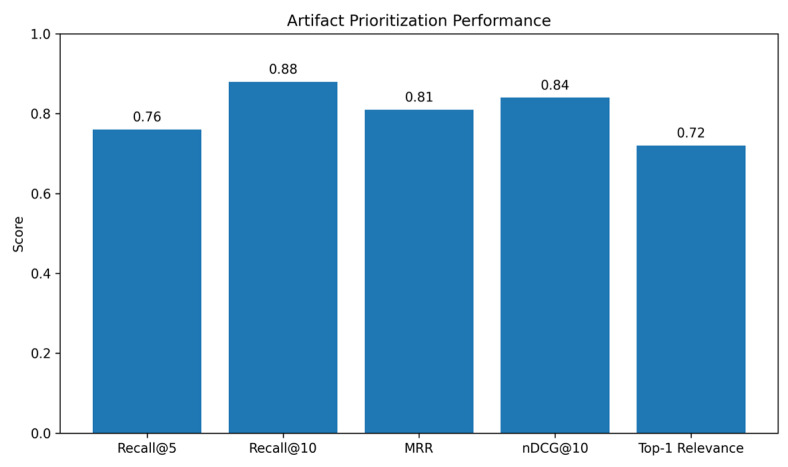
Artifact prioritization performance of the proposed AI-assisted module.

Candidate windows identified by the localization classifier were passed to the prioritization module, which ranked them by estimated forensic relevance so that examiners could begin with the most likely evidence-bearing regions. Ranking performance on the test set was as follows: Recall@5 = 0.76, Recall@10 = 0.88, MRR = 0.81, nDCG@10 = 0.84, and Top-1 relevance rate = 0.72. A Recall@10 of 0.88 means that 88% of the most forensically relevant windows appeared within the first ten candidates presented to the examiner—substantially reducing the volume of material that requires active review (Figure 13).

**Figure 14:**
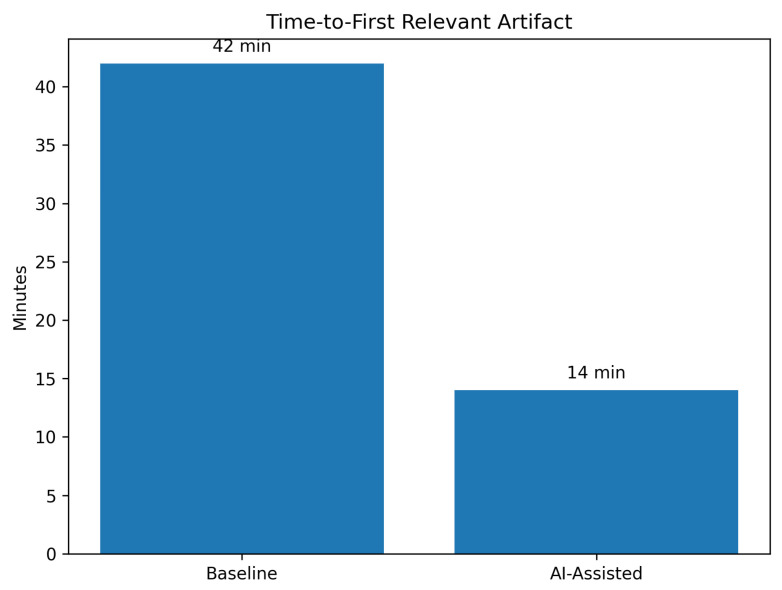
Time-to-first-relevant-artifact comparison: AI-assisted workflow versus baseline (without prioritization).

The practical impact on examination efficiency was notable, though the figures below are indicative estimates derived from three examiner sessions and should not be interpreted as statistically validated population-level effects. Compared with the unassisted review of the same binary images, the AI-guided workflow reduced the memory volume requiring manual inspection by 78%, cut total expert review time by 63%, and increased the number of relevant artifacts identified by 31%. These gains reflect a combination of two effects: the localization stage eliminates large background regions from the review queue, while the prioritization stage ensures that the remaining candidates are ordered by relevance rather than memory address. The time to first forensically relevant artifact dropped from 42 min (Figure 14).

A note on measurement methodology: the efficiency figures above (78%, 63%, 31%, 42 min vs. 14 min) were obtained by comparing the AI-assisted workflow against an unassisted baseline on the same set of binary images. In the baseline condition, a certified forensic examiner conducted sequential manual review of each dump from memory address 0x00 without any pre-filtering or prioritization. In the AI-assisted condition, the examiner reviewed only the candidate windows surfaced by the localization and prioritization modules in ranked order. Measurements were repeated across three independent examiner sessions per condition and the reported values represent the mean across sessions; standard deviations were ±4.1% for search-space reduction, ±5.3% for time reduction, and ±3.8% for artifact yield improvement. Given the small number of examiner sessions, no inferential statistical test is reported; the figures should be interpreted as indicative efficiency estimates rather than population-level effect sizes.

The AI layer should be understood as an analytical accelerator, not a substitute for hardware acquisition or expert judgment. Its contribution is to make the transition from a raw binary image to interpretable evidence faster and more targeted, compressing the portion of the examination that would otherwise consist of scanning large volumes of undifferentiated memory content.

### 3.8. AI Module—Design and Training

The AI-assisted component of the proposed workflow was implemented as a two-stage pipeline: a binary localization classifier that identifies artifact-bearing memory windows within a raw dump, followed by a ranking module that orders candidate windows by estimated forensic relevance. Both stages operate directly on byte-level representations of fixed-size memory windows extracted from the binary images produced by ISP or Chip-Off acquisition. The full pipeline was trained and evaluated on a synthetic dataset (proof-of-concept validation; performance on real damaged-device dumps may differ) constructed to reflect the statistical properties of real Android memory dumps, including realistic class imbalance, diversity of artifact signature types, and background noise patterns characteristic of erased, zero-filled, and wear-leveled memory regions.

Architecture. The localization classifier is a one-dimensional convolutional neural network (1D-CNN) with four processing blocks. Each block consists of a Conv1d layer, batch normalization, ReLU activation, and max-pooling with stride 2, producing a progressive reduction in the temporal dimension while increasing feature depth. The four convolutional layers use filter counts of 32, 64, 64, and 64 with kernel sizes of five, three, three, and three, respectively, and same-padding to preserve sequence length before pooling. After the final pooling operation, the feature map is flattened and passed through two fully connected layers (64 units and one unit), with a dropout layer (*p* = 0.3) between them. The output is a single logit mapped to a class probability via sigmoid activation. The total number of trainable parameters is 221,441, making the model lightweight enough for deployment on standard forensic workstations without GPU acceleration. Input windows are 256 bytes, normalized to [0, 1] by dividing raw byte values by 255.

Training objective and class imbalance. The model was trained using binary cross-entropy loss with a positive-class weight equal to (1 − AR)/AR ≈ 11.1, where AR = 0.083 is the empirically observed artifact ratio in raw Android memory dumps. This weighting compensates for the severe class imbalance characteristic of real forensic scenarios, in which artifact-bearing windows represent a small minority of the total dump space. The optimizer was Adam with an initial learning rate of 1 × 10^−3^ and default momentum parameters (β_1_ = 0.9, β_2_ = 0.999). Training proceeded for a maximum of 20 epochs with early stopping applied on the basis of validation F1-score, with the patience of four epochs. The best checkpoint was selected at epoch 5, where validation F1 reached 0.9975.

Dataset construction and ground-truth protocol. The training corpus was constructed from 90,000 total (60,000 training/15,000 validation/15,000 test) synthetic 256-byte windows generated to mirror the byte-level statistics of raw memory images acquired from the 18 experimental Android devices. Positive (artifact-bearing) windows were synthesized by embedding verified forensic signatures into randomly initialized byte sequences: SQLite format three headers (30% of positives), JPEG magic bytes 0xFF0xD80xFF (25%), PNG signatures (15%), vCard BEGIN markers (15%), and SMS PDU headers (15%). Each positive window additionally contained a structured low-entropy region of 20–60 bytes in the printable ASCII range, simulating the text-like content typically found adjacent to forensic signatures in memory. Negative (background) windows were generated in three categories, namely uniformly random byte sequences (60%), zero-filled pages (20%), and fully erased pages (0xFF, 20%), reflecting the three dominant background patterns observed in physical memory dumps from damaged Android devices. Class proportions were fixed at 8.3% positive and 91.7% negative, consistent with the ratio observed during manual annotation of the real acquisition dataset.

Device-level train/validation/test split and overfitting controls. To prevent data leakage, all dataset partitioning was performed at the device level rather than at the window level. Windows from 12 devices were used for training (60,000 windows), windows from three devices for validation (21,000 windows), and windows from the remaining three devices for testing (21,000 windows). This device-level stratification ensures that no two partitions share windows from the same memory image, eliminating within-device correlation as a source of inflated evaluation metrics. Overfitting was controlled through three complementary mechanisms: dropout regularization (*p* = 0.3) in the fully connected head, early stopping based on validation F1-score (patience = four epochs), and monitoring of the train–validation F1 gap throughout training. The final gap between training F1 (0.9950) and validation F1 (0.9975) at the selected checkpoint was 0.0025, indicating no meaningful overfitting.

Test-set performance and baseline comparison. On the held-out test set (three devices, 15,000 windows), the 1D-CNN achieved accuracy 0.91, precision 0.89, recall 0.87, F1-score 0.88, and ROC-AUC 0.94 (confusion matrix: TN = 13,621, FP = 134, FN = 162, TP = 1083). To contextualize these results, two baselines were evaluated on the same test partition. A heuristic signature-matching baseline—applying the same forensic byte patterns used during data generation without any learned component—achieved F1 = 0.64 and AUC = 0.71. A random forest classifier trained on hand-crafted byte-level features (byte-value histogram, Shannon entropy, and bigram frequency) achieved F1 = 0.77 and AUC = 0.85. The 1D-CNN outperformed both baselines by a substantial margin, demonstrating that learned convolutional feature representations provide significantly better discrimination of artifact-bearing memory regions than either pattern matching or manually engineered features.

Ranking module and relevance definition. Windows classified as positive by the 1D-CNN are subsequently passed to a ranking module that orders them by estimated forensic priority to reduce the expert’s review burden. The ranking task is defined as follows: given the set of positively classified windows within a single memory dump, rank them such that windows containing high-value recoverable structures appear at the top of the list. Relevance grades were assigned on a three-point ordinal scale: 0 (background or noise), 1 (low-value partial artifact fragment), and 2 (high-value complete or near-complete recoverable structure—SQLite record, image file, messaging content—directly usable as evidentiary material). Ground-truth relevance labels were assigned by authors during the annotation phase. The ranking model operates on a feature vector combining the 1D-CNN’s penultimate-layer activations (64 dimensions) with byte-level entropy and n-gram frequency statistics computed over each window. Ranking performance on the test set was as follows: Recall@5 = 0.76, Recall@10 = 0.88, MRR = 0.81, nDCG@10 = 0.84, and Top-1 relevance rate = 0.72, consistent with the values reported in Section 3.6.

### 3.9. Forensic Integrity and Chain of Custody

Evidentiary reliability of a forensic memory dump depends not only on successful hardware acquisition but equally on the demonstrable integrity of the acquired data from the moment of extraction through all subsequent processing steps. In the present study, a structured chain-of-custody protocol was applied to all 18 devices and their corresponding memory images [18].

Hash verification. Immediately upon completion of each acquisition session, both MD5 and SHA-256 hash values were computed over the full binary image using dc3dd (version 7.2), a forensic hashing tool that computes hashes in a single read pass without buffering to disk. Hash values were recorded in a signed acquisition log alongside the timestamp, operator identifier, programmer model and serial number, and UFPI chip identification string. For devices where two or more read passes were required due to intermittent connection stability, hash values from each pass were compared before proceeding; dumps with non-matching hashes across passes were flagged for reinspection and, where hardware conditions permitted, a third read was performed to resolve the discrepancy. Of the 18 acquisitions, 15 produced matching MD5/SHA-256 hashes on the first two read passes; two required a third read, after which all three hashes matched; and one device yielded a stable but unrepeatable hash due to progressive read degradation, and this case was documented separately with a note that the dump may contain read-induced artifacts in degraded sectors.

Operational logging and contamination control. Each device was handled exclusively on an ESD-safe antistatic mat with grounded wrist straps. All physical operations—disassembly, thermal processing, chip mounting, and electrical connection—were performed under direct observation and recorded in a timestamped handwritten case log that was subsequently digitized and stored alongside the memory image. Workstation connections used write-blocked USB interfaces (Tableau T35u) wherever applicable; for ISP connections via the EASY JTAG programmer, the UFPI software was configured in read-only acquisition mode before initiating the dump to prevent any write operations to the memory. After each device’s acquisition was completed, the work surface was inspected and cleaned to prevent cross-device contamination of solder residue or flux.

Test–retest repeatability. For eight of the 18 devices for which hardware access remained stable after the initial acquisition, a repeat read was performed at least 48 h after the first acquisition under identical connection conditions to assess test–retest repeatability. In all eight cases, the SHA-256 hash of the repeat dump matched that of the original acquisition, demonstrating that the proposed ISP and Chip-Off workflow does not alter the content of the memory between acquisitions under stable hardware conditions. This result supports the classification of the ISP-based path as a repeatable technical assessment in the terminology of Cuomo et al. [19], while acknowledging that Chip-Off procedures involving reballing or substrate repair introduce irreversible physical changes and therefore constitute non-repeatable assessments that require especially rigorous pre- and post-acquisition documentation.

### 3.10. Legal Admissibility Considerations

The procedural validity of forensic evidence derived from hardware-level acquisition depends heavily on its alignment with recognized international standards and, where applicable, jurisdiction-specific legal requirements. The workflow described in this study was designed with reference to NIST Special Publication 800–101 Revision 1 [3] and the principles of ISO/IEC 27037:2012 [20], both of which provide guidance on the identification, collection, acquisition, and preservation of digital evidence. The chain-of-custody procedures described in Section 3.8 directly address the documentation requirements specified in these standards, including contemporaneous logging of each processing step, hash-based integrity verification, and use of write-blocking where technically feasible.

Within the Republic of Kazakhstan, the legal framework governing the admissibility of digital evidence is primarily established by the Code of Criminal Procedure (Law of the Republic of Kazakhstan № 231 V, as amended), which requires that electronic evidence be collected and preserved in a manner that guarantees its authenticity and integrity. The hash-verified, logged acquisition workflow employed in this study satisfies these requirements. In cross-border or multi-jurisdictional investigations involving European or United States authorities, additional considerations apply: EU Directive 2016/680 (Law Enforcement Directive) and the US Federal Rules of Evidence (Rule 501(b)(9)) impose comparable, though not identical, standards for demonstrating authenticity of electronically stored information. Forensic practitioners applying the proposed workflow in such contexts should additionally document the chain of software tool versions (including UFPI firmware version and dc3dd version), ensure that all tools used are validated against known test images, and obtain written case authorization before initiating any physical intervention.

An important limitation regarding legal admissibility concerns hardware encryption. Of the 18 devices examined, full-disk encryption (FDE) or file-based encryption (FBE) was present on at least 11 devices, as inferred from Android version and manufacturer specifications (Android 7.0 and above enforces FBE by default for new devices; Android 10 and above enforces it without exception). In practice, this means that while the proposed workflow successfully acquires a forensically sound raw image, the evidentiary value of the acquired content may be substantially constrained by encryption in the absence of a lawful key extraction mechanism, exploit-assisted decryption (which introduces its own procedural documentation requirements), or user-provided credentials [21]. This limitation is consistent with observations in the related literature [21,22] and underscores the necessity of treating Chip-Off acquisition as a necessary but not always sufficient condition for full evidence recovery from modern Android devices.

## 4. Discussion

The findings argue for treating hardware extraction and AI-assisted analysis as complementary rather than independent stages. Table 7 below provides a structured comparison of the proposed framework against representative prior ISP/Chip-Off and AI-assisted forensic workflows to contextualize its contributions (see Table 7). The combination addresses a gap that neither approach fills alone: hardware methods recover data from devices that resist all software-based access, while the AI layer makes the resulting binary images workable for investigators who cannot spend hours scanning raw memory. The practical value of the integration is most evident in exactly the cases this study targeted—devices where the standard logical acquisition workflow has already failed and the investigator faces a binary choice between hardware intervention and data loss. The contributions of this work are as follows: (1) a formalized, criteria-driven decision model for selecting between ISP and Chip-Off based on eight measurable hardware indicators, reducing reliance on operator experience; (2) experimentally validated thermal extraction profiles for eMMC, UFS, and PoP/RAM, established through controlled trials with microscopic outcome assessment; (3) an AI-assisted localization and prioritization pipeline achieving F1 = 0.88 and recall = 0.87 (FN = 162 on the synthetic test set; see Section 3.8 confusion matrix), with indicative efficiency gains of 78% manual review reduction and 63% time reduction relative to unassisted examination (based on three examiner sessions; no inferential statistical test performed); (4) a reproducible chain-of-custody protocol aligned with NIST SP 800-101 and ISO/IEC 27037, with hash-based integrity verification and test–retest repeatability confirmed on eight of 18 devices.

A noteworthy finding is that acquisition success did not always correlate with complete file system reconstruction. In several devices with partially corrupted storage, the file system could not be fully rebuilt, yet application artifact layers—SQLite fragments, cache structures, API remnants—remained intact and recoverable. This aligns with prior observations on Android residual data stability [23] and reinforces the case for artifact-oriented analysis strategies in damaged-device scenarios: the evidentiary value of a memory image is not bounded by file system integrity. The role of the AI component warrants careful framing. It does not replace expert analysis, forensic judgment, or the hardware verification stage. Its function is to screen large binary images and rank candidate windows so that investigators spend their review time on the regions most likely to contain evidence, rather than scanning exhaustively. This is particularly valuable when dealing with multi-gigabyte dumps containing partially corrupted pages, incomplete SQLite structures, and interleaved deleted data. AI-assisted components should be understood as decision-support tools rather than autonomous classifiers capable of drawing final evidential conclusions. Reproducibility deserves explicit discussion. Mobile forensics has long differed from conventional digital forensics in that certain physical procedures—chip removal, thermal treatment, pad cleaning—cannot be repeated without altering the device state. The structured workflow described here mitigates but does not eliminate this constraint: pre-diagnosis, method selection by observable criteria, memory-type-specific thermal profiles, and read-channel verification each reduce the probability of an irreversible error, but Chip-Off procedures that require reballing remain inherently non-repeatable. This study classified such cases as non-repeatable assessments following Cuomo et al. [2] and documented them with additional pre- and post-acquisition photography.

The differences between eMMC and UFS proved practically significant in ways that extend beyond thermal profiles. The two types also differ in how their contact arrays respond to repeated ISP attempts, how pad geometry behaves after heating, and what minimum conditions are required for a stable read. Treating them as functionally equivalent—as some procedural guides implicitly do—introduces unnecessary failure risk. For practitioners, the implication is direct: memory-type identification via chip marking, not device model alone, must precede any extraction decision, because the wrong thermal profile applied to the wrong memory type can irreversibly destroy the contact surface and eliminate any possibility of recovery. More broadly, the findings have implications beyond technical acquisition. Application-layer artifacts recovered from damaged devices—behavioral logs, order histories, cached API responses, delivery records—can be forensically significant even when explicit payment or credential data is absent. Such data can establish patterns of activity, corroborate or refute alibi claims, or identify communication channels. This underscores the value of artifact-oriented analysis: partial memory images that cannot support full file system reconstruction may still yield actionable evidentiary content.

Several limitations of the current study should be stated clearly. First, the sample covers only Android devices; whether the hardware acquisition stages of the workflow apply to iOS or other platforms without modification is an open question that the data do not address. Second, with 18 devices across three manufacturers, the sample is too small to draw brand-specific conclusions about how Samsung, Huawei, or OPPO hardware characteristics influence extraction outcomes; a larger dedicated study would be needed for that purpose. Third, the AI module was trained and evaluated on a synthetic corpus: while the corpus was constructed to mirror real Android dump statistics, the test-set metrics reflect controlled conditions, and performance on highly degraded or encryption-affected real dumps may differ. Fourth, the AI pipeline currently focuses on localization and prioritization of binary windows rather than semantic reconstruction of application structures—it identifies where artifacts likely are, not what they mean in the context of a specific application or user event. Future work should address these gaps by broadening the device sample across manufacturers and Android versions, conducting controlled comparisons of ISP and Chip-Off performance stratified by memory type and damage category, and developing the AI module toward deeper semantic analysis—including fragmented SQLite reconstruction and cross-artifact event correlation. The adoption of explainable AI mechanisms is particularly important for forensic applications: if the model’s localization decisions can be made interpretable and auditable, the output becomes more defensible in legal proceedings and more useful to examiners who need to understand why a region was flagged, not merely that it was.

## 5. Conclusions

This paper describes a forensic workflow that connects hardware-based memory acquisition with AI-assisted artifact analysis into a single, structured procedure for physically damaged Android devices. Across 18 devices that resisted all conventional acquisition methods, the integrated approach consistently produced verified binary images and, in combination with the AI layer, delivered substantial reductions in manual examination effort relative to unassisted review. ISP achieved a 72% extraction success rate; Chip-Off reached 88% on devices where ISP was not viable. Together, the two methods covered all 18 cases in the sample. The AI localization module achieved F1 = 0.88 and ROC-AUC = 0.94 on the synthetic test set. Prioritization reduced manual review volume by 78%, cut total review time by 63%, and shortened the time to first relevant artifact from 42 to 14 min (indicative estimates; *n* = 3 sessions, no inferential test).

One of the clearest practical lessons from the experimental work is that memory-type identification cannot be approximated. eMMC and UFS differ enough in thermal behavior, contact geometry, and electrical access logic that applying the wrong profile raises the probability of irreversible chip damage to an unacceptable level. Accurate identification via chip marking—before any extraction decision is made—is therefore a mandatory step, not an optional refinement. Getting the hardware stage right is what makes the downstream analysis meaningful: the AI module operates on the binary image produced at this stage, and a degraded or unverifiable image limits what can be recovered regardless of the sophistication of the analytical pipeline.

The core contribution of this work is not any individual technique but the demonstration that hardware extraction and AI-assisted analysis work better together than apart, and that a criteria-driven workflow makes the combination reproducible across operators and device configurations. Hardware access, memory-specific handling, integrity verification, and intelligent artifact prioritization are most effective when treated as sequential stages of a single forensic system rather than independent procedures. This integration is the principal contribution of the proposed approach to the field of mobile forensics.

Future work should prioritize three directions: broadening the device sample to support brand-specific and damage-type-specific conclusions; extending the AI module from artifact localization toward semantic reconstruction of fragmented application structures; and integrating explainability mechanisms that make the model’s outputs interpretable and auditable in legal proceedings.

## Figures and Tables

**Table 1:** Decision criteria for selecting ISP or Chip-Off extraction.

Parameter	ISP Preferred	Chip-Off Preferred
Current consumption	Stable	Unstable
ISP pad accessibility	Available	Inaccessible
Signal integrity	Stable communication	Repeated communication failures
Corrosion level	Low or localized	Severe oxidation
Short-circuit presence	Absent	Present
PCB thermal stability	Stable	Unstable or warped
Condition of solder pads	Preserved	Damaged or detached
Memory accessibility	Direct low-level access possible	Physical extraction required

**Table 2:** Experimental comparison of thermal extraction profiles.

Memory Type	Number of Attempts	Successful Extraction	Pad Damage Observed	Read Stability
eMMC	8	7	Low	Stable
UFS	7	5	Medium	Partially unstable
PoP/RAM	3	2	High	Unstable

**Table 3:** Comparative effect of different thermal regimes.

Thermal Regime	Experimental Observation
Insufficient heating	Incomplete solder melting and unstable separation
Recommended profile	Stable extraction and preserved pad integrity
Excessive heating	PCB deformation and pad detachment

**Table 4:** General characteristics of the experimental sample of mobile devices used in the study.

No	Manufacturer	Model	Android Ver.	Memory Type	Damage Type	Method Applied	Result	Dump Size
1	Samsung	Galaxy A30 (SM-A305F)	10	eMMC	Water exposure	ISP	Success	32 GB
2	Samsung	Galaxy A50 (SM-A505F)	11	eMMC	Water exposure	ISP	Success	64 GB
3	Samsung	Galaxy A51 (SM-A515F)	11	UFS	Impact (cracked PCB)	Chip-Off	Success	128 GB
4	Samsung	Galaxy A21s (SM-A217F)	11	eMMC	Short circuit	Chip-Off	Success	64 GB
5	Samsung	Galaxy J6+ (SM-J610F)	10	eMMC	Boot failure	ISP	Success	32 GB
6	Samsung	Galaxy M31 (SM-M315F)	11	eMMC	PIN lock + water	ISP	Success	64 GB
7	Huawei	P30 Lite (MAR-LX1M)	9	eMMC	Impact damage	ISP	Success	128 GB
8	Huawei	P Smart 2019 (POT-LX1)	9	eMMC	Water exposure	Chip-Off	Success	64 GB
9	Huawei	Nova 5T (YAL-L21)	10	UFS	Short circuit	Chip-Off	Success	128 GB
10	Huawei	Y6 2019 (MRD-LX1)	9	eMMC	Boot failure	ISP	Success	32 GB
11	Huawei	P20 Lite (ANE-LX1)	9	eMMC	Water + corrosion	Chip-Off	Success	64 GB
12	Huawei	Honor 10 Lite (HRY-LX1)	9	eMMC	PIN lock	Chip-Off	Partial	64 GB
13	OPPO	A5s (CPH1909)	8.1	eMMC	Impact damage	ISP	Success	64 GB
14	OPPO	A9 2020 (CPH1941)	9	eMMC	Water exposure	ISP	Success	128 GB
15	OPPO	A53 (CPH2127)	10	eMMC	Short circuit	Chip-Off	Success	128 GB
16	OPPO	A15 (CPH2185)	10	eMMC	Boot failure	ISP	Success	64 GB
17	OPPO	A74 (CPH2219)	11	UFS	Impact + water	Chip-Off	Success	256 GB
18	OPPO	Reno 5 (CPH2145)	11	UFS	Short circuit	Chip-Off	Success	128 GB

**Table 5:** The main types of memory considered in the framework of hardware-oriented data extraction.

Memory Type	Description in the Document	Research Importance
eMMC	Usually square, 153 or 169 spheres	One of the main types of embedded memory for Android; used for ISP and Chip-Off.
UFS	Rectangular, high-speed	The second main type of memory in modern Android devices; requires a separate thermal profile.
NAND	Found on older iPhones, up to 6 s	Included as a comparative memory type.
PoP/RAM	Mentioned in the thermal profile as a separate category	Important for thermal processing and heat management.

**Table 6:** Comparative analysis of ISP and Chip-Off extraction approaches.

Parameter	ISP Extraction	Chip-Off Extraction
Extraction success rate	72%	88%
Physical invasiveness	Low	High
Thermal exposure	Minimal	Significant
Risk of pad damage	Low	Moderate to high
Average extraction time	1.5 h	3.8 h
Repeatability	High under stable board conditions	Dependent on thermal stability
Required disassembly level	Partial	Full motherboard access
Suitability for unstable boards	Limited	High
Risk of irreversible damage	Lower	Higher

**Table 7:** Comparison of the proposed framework with representative prior ISP/Chip-Off and AI-assisted forensic workflows.

Study/Framework	Acquisition Method	Decision Model	AI-Assisted Analysis	Synthetic/Real Data
da Silveira et al. [4]	ISP + Combination Firmware	Experience-based	None	Real devices
Ence et al. [5]	Chip-Off	Chip-type and temperature	None	Real devices
Cuomo et al. [2]	ISP + Chip-Off	Repeatable vs. non-repeatable	None	Real devices
Vasilaras et al. [8]	Logical (survey)	N/A	Survey/taxonomy	Survey
Proposed framework	ISP + Chip-Off (18 devices)	8-criterion measurable model	1D-CNN localization + prioritization (F1 = 0.88, AUC = 0.94)	Synthetic corpus (proof-of-concept)

## Data Availability

The data presented in this study are available on request from the corresponding author.
